# Myo-inositol concentration in the medial prefrontal cortex is associated with changes in brain white matter microstructure in early psychosis

**DOI:** 10.1038/s41398-026-04072-9

**Published:** 2026-06-02

**Authors:** Tommaso Pavan, Qiaochu Wang, Yasser Alemán-Gómez, Raoul Jenni, Martine Cleusix, Luis Alameda, Kim Q. Do, Philippe Conus, Patric Hagmann, Pascal Steullet, Paul Klauser, Lijing Xin, Ileana Jelescu

**Affiliations:** 1 Department of Radiology, Lausanne University Hospital (CHUV) and University of Lausanne (UNIL), Lausanne, Switzerland; 2 Ecole Polytechnique Fédérale de Lausanne, Lausanne, Switzerland; 3 Center for Psychiatric Neuroscience, Department of Psychiatry, Lausanne University Hospital and University of Lausanne, Lausanne, Switzerland; 4 Service of General Psychiatry, Treatment and Early Intervention in Psychosis Program. Lausanne University Hospital (CHUV), Lausanne, Switzerland; 5 Department of Psychosis Studies, Institute of Psychiatry, Psychology and Neuroscience. King’s College of London, London, UK; 6 Centro Investigacion Biomedica en Red de Salud Mental (CIBERSAM); Instituto de Biomedicina de Sevilla (IBIS), Hospital Universitario Virgen del Rocio, Departamento de Psiquiatria, Universidad de Sevilla, Sevilla, Spain; 7 Division of Child and Adolescent Psychiatry, Department of Psychiatry, Lausanne University Hospital and the University of Lausanne, Lausanne, Switzerland; 8 Center for Biomedical Imaging (CIBM), Lausanne, Switzerland; 9 Institute of Physics (IPHYS), Ecole Polytechnique Fédérale de Lausanne, Lausanne, Switzerland

**Keywords:** Schizophrenia, Diagnostic markers, Molecular neuroscience

## Abstract

Recent research highlights the critical role of white matter (WM) alterations in psychosis and schizophrenia (SZ), reporting volumetric and structural brain changes in affected individuals. In this study, we explored the role of astroglia in SZ, which is believed to play a role in white matter integrity. We investigated for the first time the associations between advanced diffusion Magnetic Resonance Imaging (dMRI) measures of WM microstructure and Magnetic Resonance Spectroscopy (MRS)-derived glial markers in 30 subjects with early psychosis (EP, mean age 24 ± 6) versus 49 healthy controls (HC, mean age 25 ± 6). We focused on two metabolites involved in glia: myo-Inositol (myo-Ins) and total Choline (tCho), measured in the medial prefrontal cortex (mPFC), relating them to quantitative dMRI metrics derived from Diffusion Kurtosis Imaging (DKI) and WM Tract Integrity-Watson (WMTI-W) biophysical model, including mean diffusivity and kurtosis, axonal water fraction and extra-axonal diffusivities in the whole white matter. Our findings reveal a difference between EP and HC in WM diffusivities, specifically in the extra-axonal parallel direction, but not in MRS metabolites. However, we found that the mPFC myo-Ins concentrations in EP are exclusively and strongly associated with proximal WM microstructure features, in the form of a positive correlation with axonal water fraction, a proxy for axonal density, and a negative correlation with extra-axonal parallel diffusivity, suggesting the white matter alterations could be linked to astrocytic changes in early psychosis.

## Introduction

Psychotic disorders, including schizophrenia (SZ), pose considerable challenges for the affected individuals, their families, and the broader community [1]. Recent research highlights the key role of white matter (WM) in this disease, reporting global structural changes across the brain of people suffering from psychosis [2, 3]. Thanks to its ability to exploit the random motion of water molecules, diffusion MRI (dMRI) allows us to explore the cellular environment and infer the microstructural properties of the underlying biological tissue in vivo [4]. In participants with psychosis, Diffusion Tensor Imaging (DTI) studies of WM consistently reported patterns of reduced fractional anisotropy (FA) and increased mean diffusivity (MD) [3, 5, 6]. These WM alterations are considered global, affecting the entire WM to varying degrees depending on the region [7], most notably in the frontal and prefrontal areas [2, 3]. One of the largest meta-analyses of schizophrenia patients vs controls reported the highest effect size in dMRI metrics for the average brain FA (*Cohen’s d* = 0.42), followed by the FA in the anterior corona radiata, and the genu and body of the corpus callosum [3]. In our recent work [8], we reproduced these high effect sizes in the same brain regions in patients with early psychosis (EP) and SZ. Additionally, we reported a decrease in Diffusion Kurtosis Imaging metrics [9] in both clinical groups as compared to age range-matched controls, and identified specific microstructure patterns of alterations using the biophysical diffusion model, White Matter Tract Integrity - Watson [10] (WMTI-W). Specifically, DKI provides complementary information about tissue heterogeneity by going beyond the Gaussian DTI approximation. DKI has been able to detect widespread WM abnormality in regions where typical DTI metrics (FA, MD) did not [11], e.g. regions with complex fibre arrangement [12], subtle abnormalities in subjects at high risk for psychosis [13] and microstructural connectivity patterns associated with processing speed deficits in SZ [14]. WMTI-W enables the estimation of compartment specific (intra- and extra-axonal) properties that are excellent proxies for intra-axonal injury, inflammation and abnormal myelin integrity [15–17]. WMTI-W improves upon more commonly found models like Free-water elimination [18] (FWE), by enabling the estimation of specific microstructural features, such as axonal density (*f*) and compartment-specific diffusivities, with fewer ad hoc assumptions and fit constraints [16, 19], resulting in increased validity [19]. For an overview of the effect of various pathological processes on DTI, DKI and WMTI-W diffusion metrics, see references [8, 15].

Thanks to the advanced dMRI techniques, in our previous work we found that WM alterations manifested predominantly in the extra-axonal space, as a significant increase in extra-axonal diffusivities, and that they were already present at the EP stage [8]. This latter finding is consistent with the literature showing that WM changes have been observed before the onset of psychosis [2, 20]. In ultra-high-risk individuals, existing WM alterations do not appear to suddenly worsen with the onset of psychosis [20]. Instead, longitudinal studies show a prematurely arrested FA increase during development [21], and a progressive reduction in WM FA in those who transitioned [22]. Moreover, these alterations were not associated with symptomatology [3, 8].

Various mechanisms have been proposed to explain WM alterations, ranging from early aberrant axonal pruning [23] and neuroplasticity [24], to redox dysregulation [25], and glutamatergic dysfunctions [26, 27]. A growing number of studies included neuroinflammation [28–30] and changes in neuroglia [23, 31] in the aetiology of psychosis. *Post-mortem* studies reported a decrease in astrocytes [32] and oligodendrocytes [33], which were estimated to be 20 to 27% lower in SZ patients than controls [34, 35]. On the other hand, in those patients who had high expression of inflammatory markers, ~40-50% of the brains exhibited increased immune activation (as measured by interleukin-6) in the dorsolateral prefrontal (PFC) and orbitofrontal cortices [36], increased astrogliosis [37] and reduced grey matter volume [38]. In-vivo dMRI studies have reported links between WM alterations (FW, free-water imaging [18], i.e., reduced tissue compartment) and increased blood inflammatory cytokines [39].

To disentangle the cell type contributions to the WM alterations in vivo, we employed ^1^H-Magnetic Resonance Spectroscopy (MRS) to specifically target glial integrity in EP. We focused on two key metabolites: Myo-Inositol (myo-Ins) as a marker of astrocytic density, and total Choline (tCho) as a marker of membrane turnover, glial and myelin integrity [40].

MRS measures free myo-Ins that is primarily found in high concentration in astrocytes. Free myo-Ins regulates cellular volume through its osmolytic action and constitutes a reservoir for the membrane phosphoinositides involved in intracellular signaling [40]. Reports indicate elevated expression of myo-Ins in astrogliosis [41], glioma [42] (independent of membrane turnover), and the myo-Ins diffusivity seems associated to astrocyte hypertrophy in mice [43]. Thus, myo-Ins has been proposed as a marker of astrocyte density [42] or integrity [44], and it was associated with inflammatory processes when increased [41].

In SZ, medial prefrontal myo-Ins is often found to be reduced with a small effect size [45] (standardised mean difference = 0.19), and these reductions are evident already in the early, untreated stages of psychosis [44]. Decreased myo-Ins has been associated with higher depressive symptoms in SZ [46] and across various psychiatric diseases [47], while an increase in myo-Ins to levels closer to HC improves general symptomatology and social-occupational functioning in first-episode SZ [44].

TCho measured by the MRS is composed of choline and phosphocholine (precursor of phospholipids) as well as glycerophosphocholine (product of phospholipid degradation). Therefore, tCho strongly correlates with cell density, making it a marker for membrane turnover [40, 48] and myelin [40]. Choline concentration is higher in glia (astro, micro, and oligodendrocyte precursor cells) than in neurons [49–51], and its diffusion has been proposed as a marker of (micro) glia morphology [51]. TCho is reported to be increased in the dorsolateral and medial PFC in SZ compared to HC [52, 53], in treatment-naïve first-episode psychosis [54], and in treatment-resistant SZ compared to non-treatment-resistant SZ and HC [55] (similarly to myo-Ins).

In this study, we aim to investigate the role of astroglia underlying WM alterations in psychosis by linking concentrations of metabolites associated with glial changes (myo-Ins and tCho) in the medial prefrontal cortex (mPFC), a key area for SZ [3], to the WM microstructure changes observed in early psychosis. To our knowledge, only one other study [46] investigated a similar association in chronic SZ, and reported that, when correlating metabolites with dMRI measures of the anterior corona radiata, myo-Ins was negatively associated with FA in both EP and HC, whereas tCho was not, and that myo-Ins was the sole metabolite associated with WM diffusion changes when accounting for other major metabolites (NAA, Glu).

Based on these findings, we anticipate finding lower myo-Ins levels in our medicated EP cohort as compared to HC. Therefore, we expect a negative association of myo-Ins with DTI diffusivities and extra-axonal diffusivities, and a positive association with kurtosis and axonal water fraction (i.e., lower glial density leads to higher extra-axonal water mobility, lower tissue heterogeneity and lower density of axons and other cellular processes).

In addition, to test the specificity of this association to myo-Ins and tCho, we examine the association with three other major metabolites in an exploratory and comparative manner: glutamate (Glu) and glutathione (GSH), which are respectively involved in glutamatergic dysfunction [26, 27] and oxidative stress [25]; and N-acetylaspartate (NAA), a marker for neural metabolism and myelin formation [56–58], which has been associated with WM alterations. Furthermore, we hypothesise that WM microstructure metrics will correlate with the glial marker rather than neuronal metabolites, since most of our previous findings indicate a marked increase in extra-axonal diffusivities [8], consistent with glial density changes that would reduce water hindrance outside the axons.

In previous work [59], we also tested the hypothesis that oxidative stress [25] estimated from blood markers could be partially responsible for the WM alteration in SZ. Here, we briefly tested this hypothesis again using direct brain MRS GSH.

Finally, we evaluated a series of confounders that could influence myo-Ins, tCho, and the other metabolite levels, and their association with WM microstructure in patients, such as treatment, illness duration, or substance use (e.g., cannabis). For instance, myo-Ins was investigated as a depression marker in SZ [46] following reports of lower myo-Ins levels in patients suffering from depression [60]. On the other hand, cannabis use in early psychosis is associated with reduced glutamate levels in the prefrontal cortex [61], and lower myo-Ins was related to greater problematic drug use and severity of cannabis dependence [62].

## Methods

### Participants

Data were collected from 79 individuals (Table 1) divided into two groups: 49 healthy controls (HC) and 30 participants with a diagnosis of EP, i.e., individuals within 5 years [8, 59, 63, 64] of having met the psychosis threshold according to the Comprehensive Assessment of At-Risk Mental States [65] (CAARMS). EP subjects were recruited from the Treatment and Early Intervention in Psychosis Program [66, 67] (TIPP, University Hospital of Lausanne, Lausanne, Switzerland), and included individuals suffering from non-affective and affective psychosis (n = 8, 26%). Utilising the CAARMS is standard practice within the TIPP program, as it provides a precise boundary for the classification of EP patients, and ensures enough discriminant power between patients with attenuated psychotic symptoms and those with psychosis, thereby preventing the erroneous inclusion of ultra-high risk individuals in the EP group [68]. Subjects with psychosis related to intoxication or organic brain disease, IQ < 70, reporting alcoholism, drug abuse, major somatic disease, or documented anamnestic or current organic brain damage were excluded. HC participants were recruited from the same sociodemographic area as the patients. HC participants were excluded if they or a first-degree family member reported having suffered from neurological, traumatic, major mood disorder, psychosis, prodromal symptoms, or current or past antipsychotic treatments. Diagnoses are reported in Table S1. The study was approved by the local Ethics Committee (CER-VD 382/11 and CER 13/08). Informed consent was obtained from all participants.

**Table 1:** Cohort demographics.

		EP (*N* = 30)		HC (*N* = 49)		EP-HC	
		Mean	Std. Dev.	Mean	Std. Dev.	t-value	p-value
Age (years)		24.58	5.27	25.58	4.93	-0.85	_
myo-Ins_mPFC_ (μmol/g)		7.91	1.13	8.23	0.94	-1.31	-
tCho_mPFC_ (μmol/g)		2.56	0.44	2.54	0.33	0.13	-
Glu_mPFC_ (μmol/g)		13.49	1.40	13.91	1.23	-1.37	-
GSH_mPFC_ (μmol/g)		1.25	0.30	1.25	0.27	0.07	-
NAA_mPFC_ (μmol/g)		12.59	1.34	12.86	0.99	-0.96	-
Brain volume (cm^3^)		1162.22	97.74	1177.89	115.43	-2.8	0.0068^†^
GM volume (cm^3^)		680.81	60.43	688.05	66.70	-1.53	-
WM volume (cm^3^)		463.37	48.51	474.83	55.33	-2.38	0.02^†^
Ventricle volume (cm^3^)		18.04	8.16	15.01	5.38	1.68	-
GAF		59.43	11.11	83.40	4.62	-11.19	<0.0001^†^
PANSS-total		62.07	16.71				
Age at Onset (years)		22.55	5.22				
Duration of illness (days)		691.77	511.49				
CPZ equivalent (mg/day)		340.59	272.49				
		N	%	N	%		
Sex	Female	10	33.3	17	34.7		
	Male	20	66.7	32	65.3		

*EP* early psychosis, *HC* healthy controls, *mPFC* medial prefrontal cortex, *GSH* glutathione, *Glu* glutamate, *Ins* inositol, *tCho* total choline, *WM* white matter, *GAF* global assessment of functioning, *PANSS* positive and negative syndrome scale, *CPZ* chlorpromazine.

^†^: brain volumes t-tests statistics were computed on the volumes normalized by the estimated total intracranial volume (eTICV).

### MRI acquisition

Data acquisition was performed as described in our previous works [8, 64]. Briefly, MRI scanning sessions were performed on a 3-Tesla system (Magnetom TrioTim, Siemens Healthineers, Erlangen, Germany), equipped with a 32-channel head coil. A 1-mm isotropic T_1_-weighted image was acquired. Whole-brain diffusion-weighted images (DWI) were acquired using a diffusion spectrum imaging (DSI) scheme across 15 b-values, ranging from 0 to 8000 s/mm^2^, with a spatial resolution of 2.2 ×2.2 ×3 mm^3^. Additional acquisition details can be found in the Supplementary Material.

### Image preprocessing

The T_1_-weighted images were bias field corrected [69], volumes were estimated with FreeSurfer [70] and normalised by the estimated total intracranial volume (eTICV). The diffusion preprocessing pipeline included MP-PCA denoising and corrections for Gibbs ringing, EPI distortions, eddy currents and motion, following the most recent guidelines [71]. See Supplementary Material for preprocessing details.

### Microstructure estimation

For DKI and WMTI-W estimation, the diffusion dataset was truncated [72] at b ≤ 2500 s/mm^2^. DKI was fit voxel-wise in the entire brain [73] using a weighted linear-least squares algorithm in Matlab [73], from which seven scalar maps were derived – four from DTI: radial, mean, axial diffusivity (RD, MD, AD) and FA, and three from DKI: radial, mean, axial kurtosis (RK, MK, AK). WMTI-W parameters were estimated voxel-wise from the seven DTI/DKI scalars, using an in-house Python script (https://www.github.com/Mic-map/WMTI-Watson_Python), yielding five parameter maps: axonal water fraction *f*, intra-axonal diffusivity *D*_*a*_, extra-axonal parallel and perpendicular diffusivities *D*_*e,||*_, *D*_*e*,⊥_ and axon orientation alignment *c*_2_. For each subject, voxels with unphysical values (RD, MD, AD > 4 µm^2^/ms; FA > 1, RK, MK, AK > 10) or negative values in any of the parameters were excluded from the analysis across all parametric maps. However, we did not observe unphysical or negative values in the skeletonised maps.

### ^1^H Magnetic resonance spectroscopy

All MRS measurements were performed on a 3 T Trio MR scanner (Siemens Medical Solutions, Erlangen, Germany) with a TEM volume coil. B_0_ field inhomogeneity was optimised using 1^st^ and 2^nd^ order shimming with FAST(EST)MAP [74]. ^1^H-MR spectra were obtained in the voxel located in the mPFC (Fig. S1 in the supplementary material) using the SPECIAL [75] localization sequence (TE/TR = 6/4000 ms, VOI = 20×20×25mm^3^, 148 averages). Water unsuppressed spectra were acquired with the same parameters (2 averages) as an internal reference.

### Spectral quantification

Metabolite concentrations were quantified with LCModel [76] using unsuppressed water MR spectra as an internal reference. Tissue composition within the MRS voxel was evaluated based on the segmentation of 3D MPRAGE images. Fractions of white matter (WM), grey matter (GM) and cerebrospinal fluid (CSF) were used to correct for water content in the measurement voxel. The GSH and Glu levels were reported previously [77]. The MRS voxels only partially overlapped with the WM and contained primarily GM, together with some WM and CSF fractions. There was no overlap between the MRS voxel and the WM once skeletonised.

### Tract-based spatial statistics

Associations between MRS metabolite concentrations and dMRI microstructure metrics were tested at the voxel level using FSL’s Tract-Based Spatial Statistics [78] (TBSS). First, individual FA maps were used to build a study-specific FA template using ANTs [79]. Then, the estimated warps were applied to each of the 12 dMRI parametric maps for spatial normalisation. From the average FA, the WM skeleton mask (FA > 0.25) was estimated and used for permutation testing (FSL randomise [80], 5000 permutations) on all dMRI metrics. The resulting statistical maps were corrected using family-wise error rate (FWER) and Threshold-Free Cluster Enhancement (TFCE). Significant voxels were averaged and plotted alongside the cluster maps for each significant contrast. Additionally, we report only the associations with average cluster p-value < 0.0083 (0.05/6 contrasts). The relationship between metabolite concentration and each microstructure metric was modelled as

1 $$E\left(V\right)={\beta }_{0}+{\beta }_{1}M+{\beta }_{2}{Diagnosis}+{\beta }_{3}M\times {Diagnosis}+{\beta }_{4}{Age}+{\beta }_{5}{Sex}$$

where $E\left(V\right)$ is the expected value of a voxel, and *M* is one of the metabolites of interest. A correction for age and sex was included systematically.

### Group, interactions, and post hoc analyses

Group and interaction analyses were carried out using linear regressions and controlled for multiple comparisons with the False Discovery Rate (FDR) correction. To verify if the other metabolites could contribute or suppress the associations within the myo-Ins clusters, we re-computed the association between myo-Ins and each microstructure metric in the significant clusters combined, identified with TBSS at the previous step, by regressing myo-Ins together with the concentrations of the other metabolites:

2 $$E\left({V}_{{\rm{myo}}-{\rm{Ins\; cluster}}}\right)={\beta }_{0}+{\beta }_{1}{Myo\mbox{-}Ins}+{\beta }_{2}{tCho}+{\beta }_{3}{Glu}+{\beta }_{4}{\rm{GSH}}+{\beta }_{5}{NAA}$$

Finally, we investigated via linear models whether myo-Ins is influenced by diagnosis (affective vs non-affective psychosis), treatment, illness duration, or substance use (e.g., cannabis, Table S2 in supplementary material). For details, see Post Hoc Analyses Methodology and Fig. S5 in the Supplementary Material.

## Results

Summary demographics of the cohort can be found in Table 1. EP (mean 24 ± 6 y/o, median 24.0 y/o) and HC (mean 25 ± 6 y/o, median 24.7 y/o) did not differ in age (p = 0.40). EP participants showed lower normalised total brain volume (p = 0.0068) and lower normalised white matter volume (p = 0.020) than HC. Functioning levels as measured by the Global Assessment of Function (GAF) were lower in EP than HC (p < 0.0001). Noteworthy is an imbalance between males ( ~ 66%) and females ( ~ 34%) among both EP and HC participants.

### Group differences in metabolites and WM microstructure

We did not find any difference between EP and HC in any metabolite concentrations (p > 0.17, Table 1). TCho (p_FDR_<0.0001, Fig. S2D) and NAA (p_FDR_=0.006, Fig. S2E) were positively associated with age, but only tCho showed differences between EP and HC in the age effect (age × diagnosis interaction, p = 0.017) before FDR correction (p_FDR_=0.084, Fig. S3D). Considering these findings and the sex imbalance, we systematically included a correction for age and sex in the TBSS analyses. Metabolite concentrations were not associated with the current dose of antipsychotic medication (chlorpromazine equivalent, p > 0.12).

Differences between EP and HC in the WM microstructure were reported in our previous work [8] in a larger cohort of which the current one is a subset (restricted to participants with both dMRI and MRS data available). Briefly, we found increased DTI diffusivities and reduced kurtosis in EP vs HC (mean |*Cohen’s d |* = 0.46). WMTI-W attributed the findings primarily to reduced axonal water fraction, *f*, and changes in the extra-axonal compartment, evident from the higher extra-axonal parallel (*D*_*e*,||_) and perpendicular (*D*_*e*,⊥_) diffusivities in EP compared to HC. In the current smaller cohort subset, we only found significantly higher MD, AD, and *D*_*e*,||_ (p < 0.035).

### Associations between myo-inositol and total choline concentrations and WM microstructure

Several clusters of associations were found in WM. Overall, we found that myo-Ins showed the largest clusters of association with the WMTI-W metrics, but not with DTI or DKI metrics (except for axial diffusivity, AD). No associations with tCho were found.

#### Myo-Inositol

**Fig. 1:**
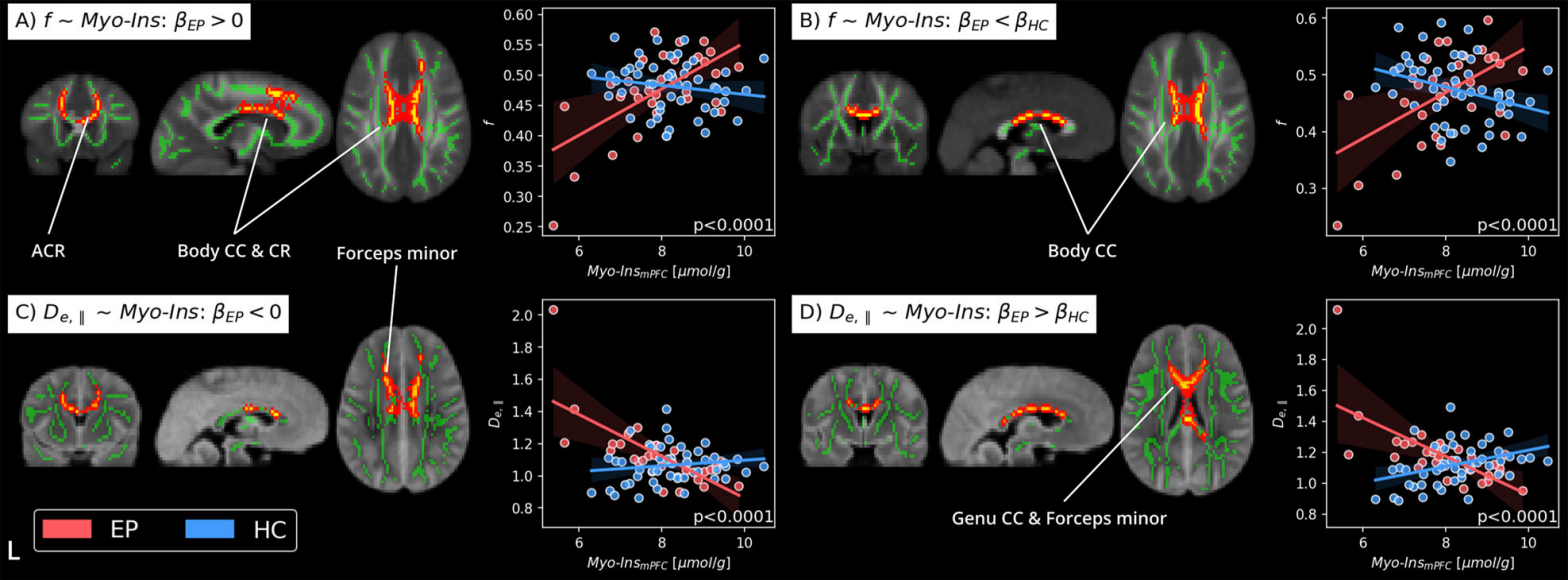
TBSS results of the associations between mPFC myo-inositol and WM microstructure metrics: axonal water fraction (*f*) and extra-axonal parallel diffusivity (*D*_*e*,||_). Significant clusters were identified in the genu and body of the corpus callosum (CC), parts of the anterior corona radiata (ACR), and the forceps minor. **A, C** In the EP group, lower myo-inositol concentrations were associated with lower axonal water fraction (*f*) and higher extra-axonal parallel diffusivity (*D*_*e*,||_). **B, D** Scatter plots showing that the slopes in the EP group differed significantly from the HC group. No significant relationship was found between myo-inositol and WM metrics in the HC group. Note, removing extreme values (*f* < 0.35 and *D*_*e*,||_ >1.6) did not change the results qualitatively. β_EP_: slope of the EP group; β_HC_: slope of the HC group. L: Left hemisphere.

Myo-Ins showed the widest and strongest association with dMRI metrics in EP. WMTI-W revealed a positive association of myo-Ins with the axonal water fraction, *f* (p < 0.0001, Fig. 1A), and a negative association with the extra-axonal parallel diffusivity, *D*_*e,||*_ (p < 0.0001, Fig. 1C) in EP, with significantly different slopes from HC (*f*, *D*_*e,||*_: p < 0.0001, Fig. 1B, D). Clusters were located primarily in the forceps minor (Fig. 1C), genu and body of the corpus callosum (Fig. 1; Hofer and Frahm [81]: region *I*-prefrontal, *II*-premotor and supplementary motor, and *III*-motor), and to a lesser extent the anterior corona radiata (Fig. 1A). Myo-Ins was also negatively associated with AD (p < 0.0001, Fig. S4), but only in the left internal capsule.

### Post Hoc Analysis

**Fig. 2:**
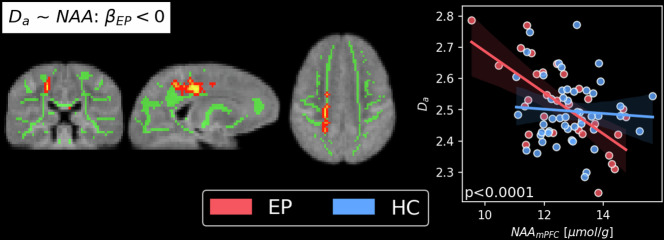
TBSS results of the associations between mPFC NAA and WM microstructure metric: intra-axonal diffusivity (*D*_*a*_). A significant cluster was found in parts of the left superior and posterior corona radiata, where *D*_*a*_ was higher at lower concentrations of the mPFC NAA in EP participants. In HC, no relationship with NAA and the WM metrics was found, and the slope of EP did not significantly differ from HC. β_EP_: slope of the EP group.

When investigating the association of the other metabolites (NAA, GSH, Glu), only one small cluster of WM microstructure association with the NAA concentration was found. Higher NAA levels were associated with lower *D*_*a*_ (p < 0.0001) in parts of the left posterior, superior corona radiata, and a smaller one in the splenium of the corpus callosum (Fig. 2). We did not find associations with GSH or Glu.

**Fig. 3:**
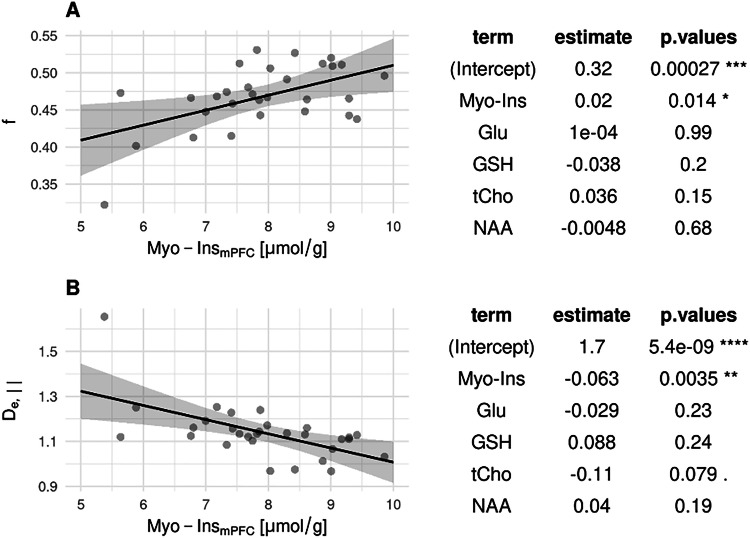
Association of EP’s WM microstructure values in the post hoc cluster with all the metabolite concentrations. **A** For axonal water fraction (*f*), myo-Inositol drives most of the associations, and its effect is not suppressed when controlling for Glu, GSH, tCho, and NAA (see Eq. 2). **B** For *D*_*e*__*,||*_ myo-Ins also drives the association regardless of other metabolites. Adjusted-R^2^: *f* = 0.23 and *D*_*e,||*_ = 0.36.

The frequency mask of the combined WM clusters associated with myo-Ins concentration (Fig. S5) showed spatial proximity to the MRS voxel in the mPFC, indicating regional specificity of WM microstructure changes to myo-Ins levels. When the EP’s average cluster mask values were regressed with all metabolites together, the myo-Ins association was not suppressed, confirming the specificity of myo-Ins concentration correlating with WM microstructure in EP: in the *f* regression model (Fig. 3A), myo-Ins was the sole significant metabolite (p < 0.014, *f* adjusted-R^2^ = 0.23). In the *D*_*e,||*_ model (Fig. 3B), myo-Ins always drove the association (p < 0.0035, *D*_*e,||*_ adjusted-R^2^ = 0.36). Coefficients of partial determination (partial R^2^) for each individual metabolite can be found in the supplementary Table S3.

Furthermore, to ensure robustness of our findings, we repeated the post hoc analyses excluding the EP participants with the most extreme datapoints (*f* < 0.35 and *D*_*e,||*_>1.6 µm^2^/ms, respectively removing 2 and 1 EP participant datapoints). Removing extreme values did not suppress the significant associations (p < 0.028 for *f* and *D*_*e,||*_ slope and group interactions; Fig. S6). Excluding female EP participants (who are the minority) did not suppress the myo-Ins association with dMRI metrics either (p < 0.00021 for *f* and *D*_*e,||*_ slope and group interactions; Fig. S7).

We also tested whether the myo-Ins and microstructure association was dependent on the inclusion of participants suffering from affective psychosis. Excluding patients (n = 8, ~26%) with such a diagnosis did not suppress the significant associations with both *f* (p_FDR_<0.006, Fig. S8A) and *D*_*e,||*_ (p_FDR_<0.019, Fig. S8B).

The results for the analyses, including medication, drugs or cannabis exposure, are reported in the Post Hoc Analyses Sections in the Supplementary Material. In short, none of the confounding variables influenced the relationship of WM microstructure (*f*, *D*_*e,||*_) with myo-Ins.

## Discussion

In this work, we linked the mPFC myo-Ins and tCho concentrations, which are glial markers [82], to the WM microstructure features in EP. Myo-Ins concentration in the mPFC showed the strongest and largest association clusters to the WM microstructure among all investigated metabolites. In EP, myo-Ins correlated positively with axonal water fraction, *f*, while large clusters of negative associations were also found with *D*_*e,||*_. However, we did not find such associations in HC nor with tCho.

In an exploratory fashion, we also investigated associations with three other major metabolites known to be involved in schizophrenia, Glu [26], GSH [25] and NAA [83], but we found limited associations with the microstructure, except for a negative association between NAA concentration and *D*_*a*_ in EP, confirming myo-Ins as the most prominent metabolite associated with WM alterations in EP. Finally, we verified that our findings were not influenced by indirect effects of treatment, substance exposure [61, 62] or other demographic characteristics.

Microstructure metrics are considered sensitive to myelination but also to glial cell density [15] as increases or decreases in tissue cellularity could hinder or facilitate water movement, respectively, altering the diffusivity properties of the WM [15, 84]. In cuprizone-intoxicated mice [84], the initial acute inflammation sharply lowers extra-axonal diffusivities ( ↓ *D*_*e,||*_, ↓ *D*_*e*,⊥_) due to cellular crowding of the extra-axonal space as a result of gliosis. Once acute inflammation subsides, demyelination dominates, reducing the axonal water fraction ( ↓ *f*) while raising diffusivities ( ↑ *D*_*e,||*_, ↑ *D*_*e*,⊥_), relative to controls. Furthermore, longitudinal validation studies related reduced parallel diffusivity to astrogliosis [84–86], and a diffusion-weighted MRS study using the same model of cuprizone intoxication showed that myo-Ins diffusivity rises with histologically confirmed astrocyte hypertrophy [87]. Together, these findings suggest that astrocytic changes directly influence diffusivity metrics.

Contrary to our expectations, the average myo-Ins concentrations in the EP group did not differ from those of HC in our cohort. In their review, Das et al. reported that in studies with a higher number of female patients, the myo-inositol reduction was much more pronounced [45]. Thus, the absence of such group differences in metabolite concentrations in our study may be due to the sex imbalance in our EP cohort (20 male vs 10 female), but our power to test sex × diagnosis × metabolite interactions was limited.

However, the TBSS analysis (corrected for age and sex) showed that participants with the most altered WM (estimates further away from HC, Fig. 1A, C) had the lowest mPFC myo-Ins levels. Low myo-Ins may indicate a reduction in astrocyte density, integrity (e.g. atrophy [32, 88]) or deficits in astrocyte activation and recruitment [45] in the mPFC, which may extend beyond that area to neighbouring WM tracts. The main clusters of associations included the anterior corona radiata, forceps minor, and the genu and body of the corpus callosum consistently, all white matter regions and fibre bundles adjacent to, or with projections to and from the mPFC, the target area of the MRS voxel. This suggests that mPFC myo-Ins concentrations are related to the WM microstructure both locally (within the MRS voxel: forceps minor) and proximally (adjacent or connected to the MRS voxel: corpus callosum body and corona radiata). Notably, the anterior corona radiata and corpus callosum have consistently been identified as among the most affected regions in SZ [3, 8] and early-onset SZ [89].

Relative to distribution values in HC, only a subset of EP patients displays lower myo-Ins, lower *f* and higher *D*_*e,||*_, which may constitute a patient subgroup. In a meta-analysis by Das et al. [45], the authors reported a small but statistically significant reduction in medial prefrontal myo-inositol levels (effect size = 0.2) in individuals with SZ. In light of the small effect size, the authors proposed that the biological pathways affecting the myo-inositol and astroglia were likely to operate only in a subset of patients with schizophrenia [45]. Remarkably, our findings strongly support this interpretation, suggesting that a combination of MRS mPFC myo-Ins and WMTI-W metrics could serve as a potential imaging biomarker for stratifying patients into subgroups. Similarly, glial changes are a core feature of SZ [32]. Electron microscopy studies showed dystrophic and swollen astrocytes in SZ [90]. However, GFAP-based and Nissl staining studies reported more controversial findings, indicating that the density reduction may not be extensive [32], or possibly characterise only a subset of patients [45], which seems to be the case in our cohort. For example, Trépanier et al. [91] found no consistent alterations in glial fibrillary acidic protein (GFAP) expression in their meta-analysis, a result potentially explained by the heterogeneity of the SZ population, which may comprise distinct subgroups with and without astrocytic abnormalities.

The causal relation between mPFC myo-Ins and WMTI-W *f* and *D*_*e,||*_ is complex and merits dedicated investigation beyond the current work. Here, we limit ourselves to some possible explanations: an astrocyte dysfunction more broadly affecting and propagating to the frontal WM in a subset of EP may be an interpretation for the observed association patterns. A reduction in the water hindrance due to the less dense astrocyte crowding of the WM extra-axonal space would be captured as a decrease in ( ↓ *f*), and an increase of extra-axonal parallel diffusivity ( ↑ *D*_*e,||*_) [15, 84–86]. This would also suggest that the WMTI-W parameters *f* and *D*_*e,||*_ may have higher specificity to WM alterations caused by glial changes than DKI and DTI scalars. In parallel, myo-Ins has an osmolytic [40, 92] effect (i.e., regulates cellular osmotic pressure), thus low myo-Ins may also be related to astrocyte and glia volume reduction or atrophy [32] in the WM, which has been found in the prefrontal WM of SZ specimens [32, 88], and could show similar WM patterns ( ↓ *f*, ↑*D*_*e,||*_) at low myo-Ins concentrations.

Myo-Ins levels correlating primarily with *D*_*e,||*_ rather than intra-axonal diffusivity (*D*_*a*_), further support the extra-axonal nature of these changes, corroborating our previous reports of altered extra-axonal compartment in EP and SZ [8]. Notably, the lack of correlations in the same regions for Glu and NAA, intraneural metabolites [50, 93], reinforces this interpretation.

We also reported a negative association between AD and myo-Ins in the internal capsule. Such an association may reflect the indirect effect of myo-Ins changes to the mPFC axons projecting to and from the internal capsule.

To our knowledge, this is the second work investigating the relationship of WM microstructure with myo-Ins and tCho in schizophrenia, and the first one reporting on EP together with specific WM microstructure metrics that aid the biological interpretation. In the work of Chiappelli and colleagues [94], the authors reported a negative association between FA and myo-Ins in both SZ and HC, and concluded that, among other metabolites such as NAA, Glu and tCho, myo-Ins was the only one driving the FA changes. The negative association with FA was interpreted as evidence of an effect of inflammation on WM microstructure. Here, we did not find any association with FA, and the significant associations were only in the EP group, but not in HC. Several factors may explain this discrepancy. First, we measured the myo-Ins concentration in the frontal GM-dominated area and not purely in the WM. Second, our cohort exclusively included EP instead of SZ. Third, our participants were younger (average age of ~25 vs ~39 years) with a narrower age distribution (18-36 vs 20-58 years old), although Chiappelli et al. reported no age effect on such association.

Finally, myo-inositol is implicated in depression, bipolar disorder, and in the mechanism of action of mood stabilising medications, like lithium. For instance, it is believed that lithium reduces brain myo-inositol concentrations, resulting in a downregulation of both membrane phospholipid synthesis and phosphatidylinositol-mediated signalling turnover [95, 96]. Although excluding participants with affective psychosis (n = 8, ~26%) and controlling for medication, drugs, or cannabis exposure did not suppress the association between myo-Ins and diffusion microstructure metrics, we cannot fully exclude the possibility that medication and diagnosis influenced our results, given the complexity of controlling for these covariates.

Aside from myo-Ins, the sole other metabolite we found associated with WM microstructure was NAA. In EP, NAA was found to be negatively associated with *D*_*a*_ in parts of the left superior and posterior corona radiata, so in different regions than for myo-Ins, possibly indicating an indirect relation between mPFC NAA and less complex and ramified intra-axonal space, axonal metabolism [97], or NAA diffusion [98] in the corona radiata of EP.

The lack of association between WM and tCho, Glu, or GSH does not exclude their role in SZ but suggests that they may not directly contribute to WM alteration in EP, or that our study lacks sufficient power to detect their effect. This view is supported by the age-tCho relationship and its trend difference between EP and HC, which could act as a confounder, misattributing covariance between metabolites and WM to age or vice versa.

The absence of associations with GSH is in line with our previous work on the peripheral GSH-redox system [59]. However, we did not replicate the previously reported association between GSH levels and the average generalised FA of the cingulum WM bundle [99], and improvements of the WM integrity following N-acetylcysteine treatment in PT [100]. Taken together, this suggests that the effect of GSH on white matter may be subtle, potentially requiring larger regions of interest to detect it. As such, the TBSS method may lack the sensitivity to capture this effect.

As the last set of analyses, we also tested the effects of treatment, illness duration, and substance exposure [61, 62] on our results, to verify that our findings were not influenced or caused by unaccounted confounding effects. Previous works showed that lower myo-Ins was related to the severity of drug and cannabis abuse [62], while myo-Ins levels were higher in treatment-resistant than non-treatment-resistant SZ or HC [55]. However, we found no association with antipsychotic medication, illness duration or substance exposure, indicating that the links between myo-Ins concentrations and WM microstructure in neighbouring tracts are independent of these factors.

### Limitations

The first limitation of our work is the different spatial localisation of the MRS and dMRI signals. Whereas the dMRI metrics were computed and tested in the WM, our single voxel MRS acquisition was centred on the mPFC and contained primarily GM together with some WM and CSF fractions. However, the significant clusters were spatially coherent with the location of the MRS voxel, supporting a plausible microstructural–metabolic WM-GM coupling. Additionally, by employing a spatially unbiased approach such as TBSS rather than a region-of-interest method, we identified anatomically meaningful clusters aligned with the MRS voxel placement in a data-driven way, which strengthens the interpretability and validity of our findings. Furthermore, the sample size of our EP cohort is relatively small (n = 30) and featured more males (n = 20) than females (n = 10). However, excluding the female group did not change the substance of our findings. Despite a systematic correction for age and sex in our tests, and the narrow age span of our participants, we cannot fully exclude the influence of maturation and ageing on the WM microstructure and metabolite concentrations (myo-Ins and tCho increase, Glu and NAA decrease with aging [101]). Finally, despite our best efforts, the measures used for medication (chlorpromazine-equivalent dose at the time of the scan) and substance use are imperfect and may not capture the patients’ cumulative drug histories and effects.

## Conclusions

In this work, we showed for the first time the comprehensive associations between mPFC concentrations of glial metabolites and advanced dMRI WM microstructure metrics in EP. We demonstrated that lower levels of mPFC myo-inositol in patients are strongly associated with proximal changes in WM tissue diffusivities that corresponded to lower axonal water fraction and less hindered extra-axonal parallel diffusivity (higher *D*_*e,||*_, lower *f*), suggesting white matter alteration could be linked to astrocytic changes in a subgroup of early psychosis patients. Furthermore, joint assessment of myo-inositol levels and white matter microstructure metrics may serve as a potential imaging biomarker for stratifying patients into subgroups, possibly characterised by glia-driven pathophysiology.

## Supplementary information

Supplementary Material

## Data Availability

The data supporting the findings of this study are available from the corresponding author upon reasonable request. The code for WMTI-W can be found here: https://github.com/Mic-map/WMTI-Watson_Python.
