# Supplementary material for: Myo-inositol concentration in the medial prefrontal cortex is associated with changes in brain white matter microstructure in early psychosis
Source: Transl Psychiatry. 2026 Jun 2;16:393. doi: 10.1038/s41398-026-04072-9 (PMC13444330; doi:10.1038/s41398-026-04072-9)
Supplement: Supplementary file 1 [file 41398_2026_4072_MOESM1_ESM.pdf]

Myo-Inositol Concentration in the Medial Prefrontal Cortex is Associated with Changes in Brain White Matter Microstructure in Early Psychosis

Tommaso Pavan, Qiaochu Wang, Yasser Alemán-Gómez, Raoul Jenni, Martine Cleusix,
Luis Alameda, Kim Q. Do, Philippe Conus, Patric Hagmann,
Pascal Steullet, Paul Klauser, Lijing Xin, Ileana Jelescu

February 18, 2026

Supplementary Material

Methods

MRI Acquisition details

Magnetisation-prepared rapid acquisition gradient echo (MPRAGE): echo time (TE) 2.98ms, repetition time (TR) 2300 ms, inversion time (TI) 900 ms, field of view (FOV) 160 x 240 x 256 mm^3 and voxel size 1 x 1 x 1.2 mm^3), acquisition time 7 minutes. Pulsed Gradient Spin-echo (PGSE) echo planar imaging (EPI) sequence: acquired in Cartesian q-space coverage totalling 257 DWI volumes. TE = 103 ms, TR = 5.9 s. FOV = 211 x 211 x 114mm^3, voxel size = 2.2 x 2.2 x 3mm^3, 96x96x38 slices, partial Fourier = 0.75, 1 b=0 acquisition and 256 directions, acquisition time 13 minutes. Any participants with a pacemaker, cochlear implant, implant of a stimulator or drug pump, glucose sensor, bypass valve or pregnant were not allowed in the MRI scanner and excluded from the study.

MRI Preprocessing details

The diffusion preprocessing pipeline included MP-PCA denoising (Veraart et al., 2016) and Gibbs ringing correction (Kellner et al., 2016; Lee et al., 2021). The EPI distortions were corrected using ANTs non-linear registration since no reverse phase encode image or magnetic field map was available. The registration of the DSI b=0 volume to the MPRAGE was constrained only in the direction of the distortion, and the estimated correction

was warped back to each DWI volume in native space (Alemán-Gómez et al., 2023; Tax et al., 2022). Thereafter, the distortion-corrected images were further corrected for eddy currents and motion using FSL *eddy* (Andersson and Sotiropoulos, 2016). Since FSL eddy does not support DSI data natively (see *–data_is_shelled* in: https://fsl.fmrib.ox.ac.uk/fsl/fslwiki/eddy/UsersGuide), temporarily merging or directly dropping a subset of volumes based on their b-values in order to simulate shells was necessary to accommodate the algorithm. Namely, volumes b=1500 and b=2000 were merged as b=1750, b=4000 was merged to b=4500 while b=6000 and b=8000 were removed. Right after the eddy execution, the merged volumes were split back into their original b-values, except for the dropped volumes (for the dMRI metric estimations, only the b-values$\leq$2500 were needed). The reduced DWI images featured 57 directions (1:b=0, 6:b=500, 12:b=1000, 8:b1500, 6:b=2000, 24:b=2500). Data quality was assessed by visual inspection.

Post hoc analyses methodology

White matter (WM) post hoc analyses were computed from the frequency mask of the significant myo-inositol (myo-Ins) clusters (Fig. S5). The mask was generated by combining all the masks of significant WM clusters across dMRI modalities and contrasts of the myo-Ins associations.

Diagnosis

Diagnosis	N	%
Schizophrenia	16	53.3
Bipolar disorder	1	3.3
Bipolar disorder with psychotic features	1	3.3
Depressive disorder with psychotic features	1	3.3
Brief psychotic disorder	4	13.3
Unspecified psychotic disorder	2	6.7
Schizoaffective disorder	5	16.7

Table S 1: Early Psychosis patients' individual diagnoses. This includes patients from the broader psychosis spectrum, including affective psychosis.

Substance Exposure

To evaluate the effect of substance abuse, we use the Case Manager Rating Scale (CMRS). CMRS is a 10 item scale indicating the frequency of use of 10 different substances from 1 (absent) to 5 (extremely severe). The substances tracked are: Cannabis, Cocaine, Hallucinogens, Opiates, PCP, Stimulants, Sedatives/Hypnotics/Anxiolytics, Non-prescribed medications, and Others. The cumulative rating is computed by summing all the individual scales and subtracting 9 (so absent=1). Every participant with CMRS$\geq$2 (cannabis and total) was classified as exposed. Among the EP cohort, we found CMRS-Cannabis: non-exposed=14, exposed=9 subjects (mean score=2.44). CMRS-Total: non-exposed=7, exposed=16 subjects (mean score=2.48). For the specific distribution, see table S2.

CMRS score:	1 (No use)	2	3	4	5 (Extremely severe)
Alcohol	11	18	0	1	0
Cannabis	19	6	5	0	0
Cocaine	29	1	0	0	0
Hallucinogens	30	0	0	0	0
Non-Prescribed Meds	30	0	0	0	0
Opioids	29	0	1	0	0
Others	30	0	0	0	0
PCP	30	0	0	0	0
Sedatives	30	0	0	0	0
Stimulants	30	0	0	0	0

Table S 2: Count of Early Psychosis by type of substance and score.

MRS voxel placement

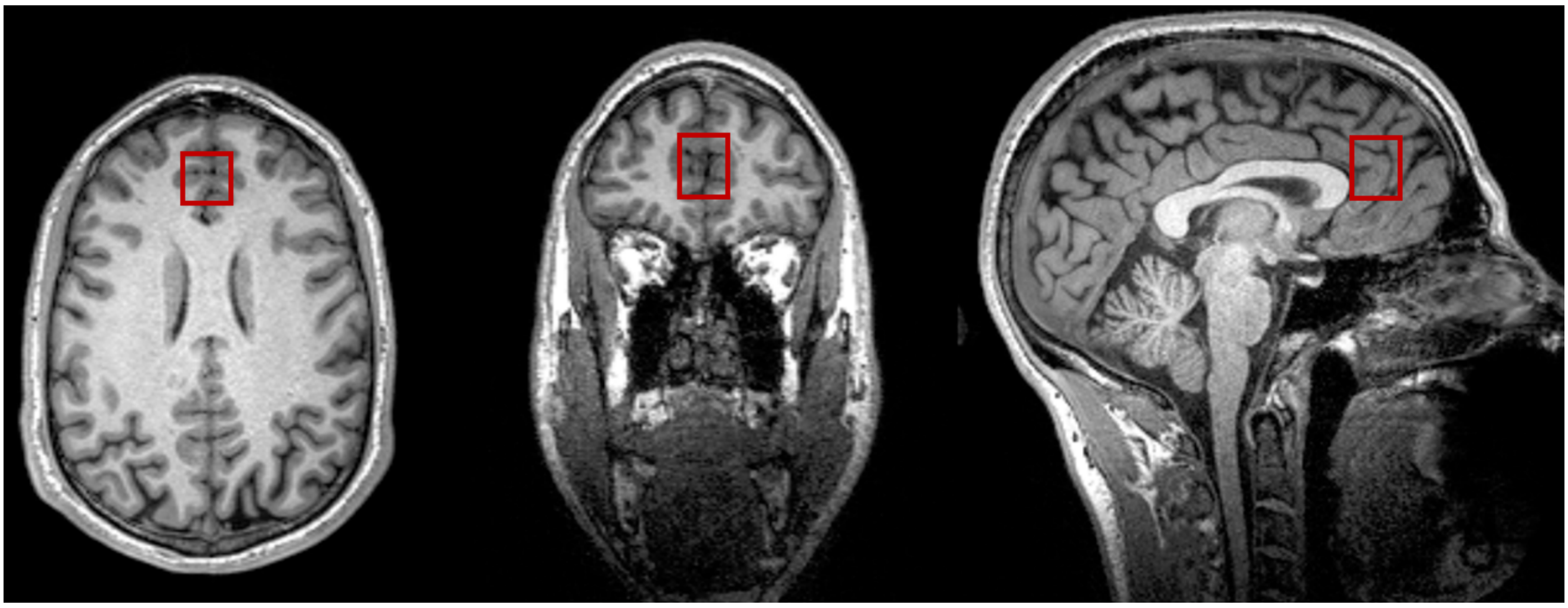

Figure S 1: Placement of the single MRS voxel in the medial prefrontal cortex (mPFC).

Supplementary results

Age affect on the MRS metabolites

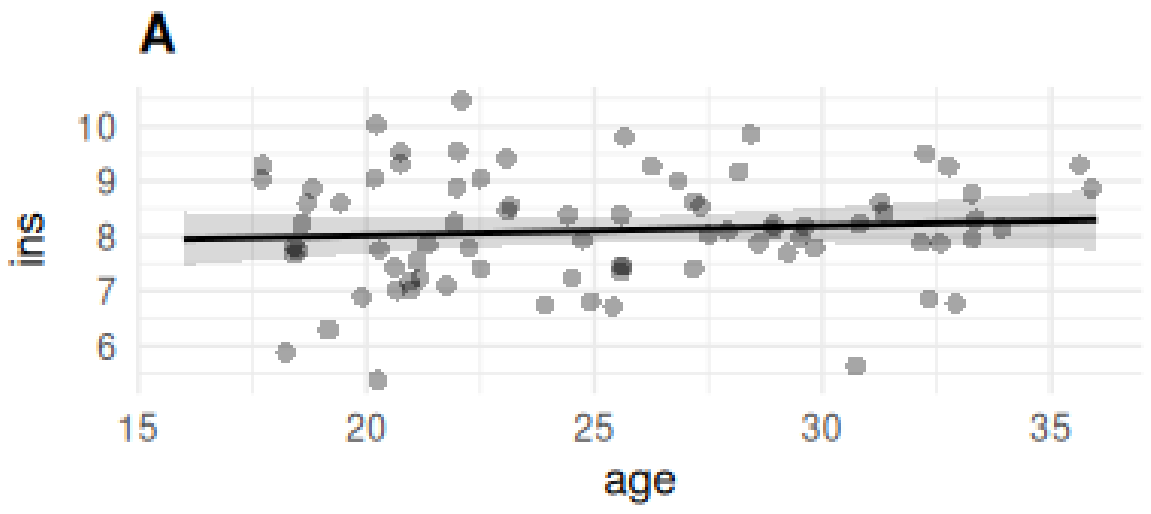

term	estimate	p.values
(Intercept)	7.7	3.5e-21 ****
age	0.018	0.45

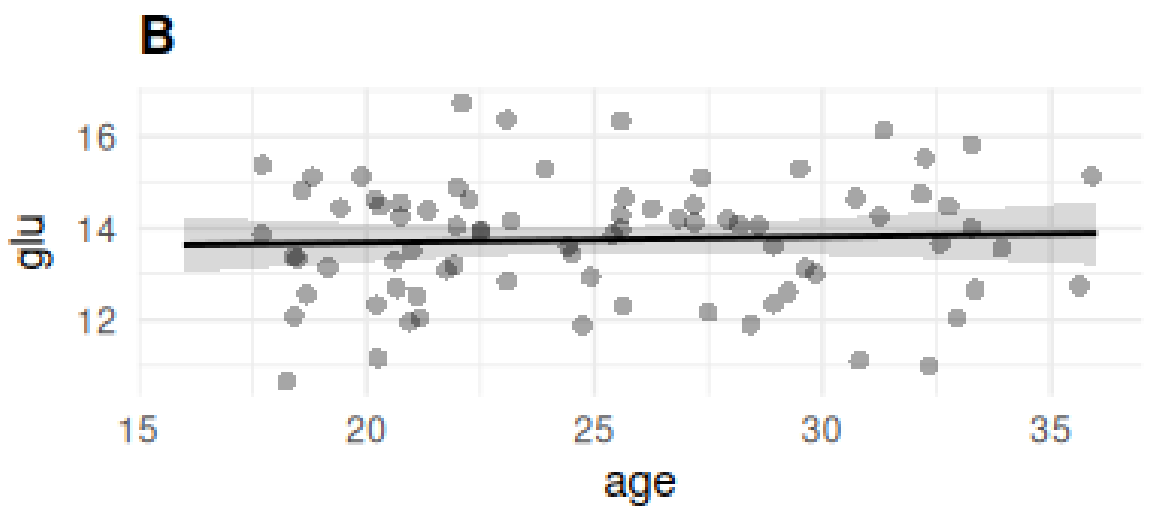

term	estimate	p.values
(Intercept)	13	5.6e-29 ****
age	0.013	0.66

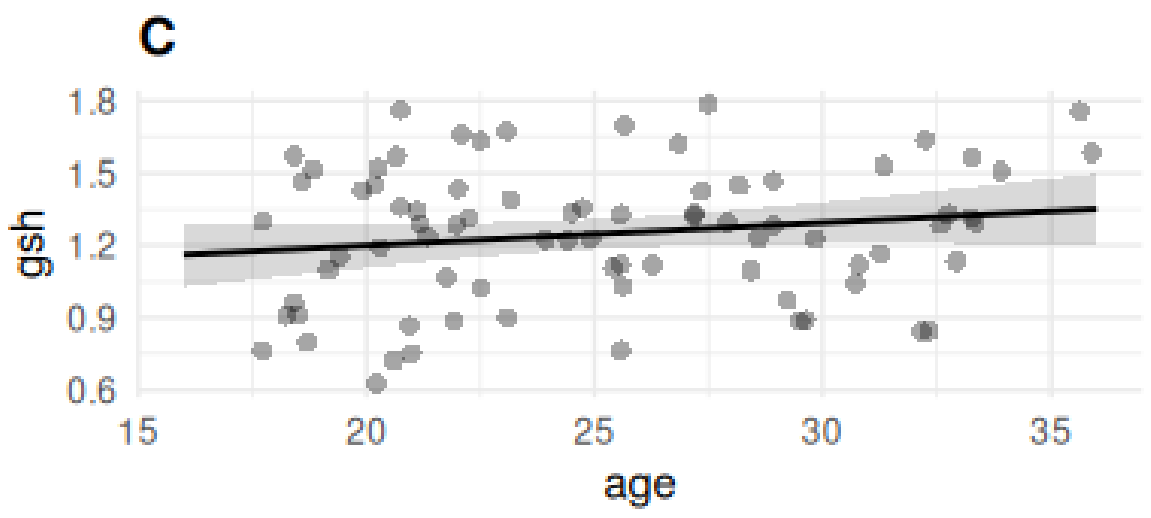

term	estimate	p.values
(Intercept)	1	1.9e-08 ****
age	0.0096	0.13

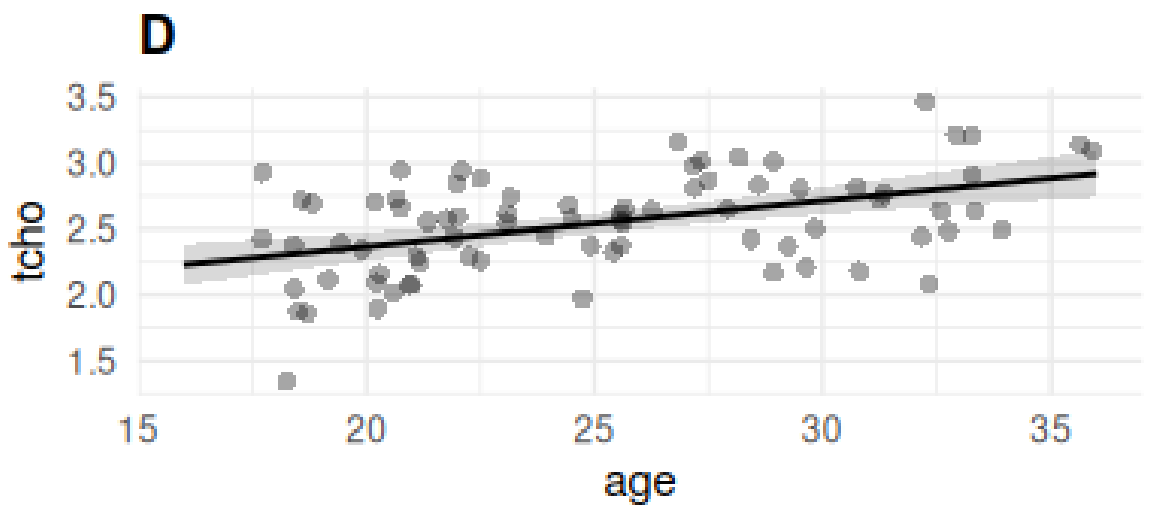

term	estimate	p.values
(Intercept)	1.7	4.2e-13 ****
age	0.034	1.7e-05 ****

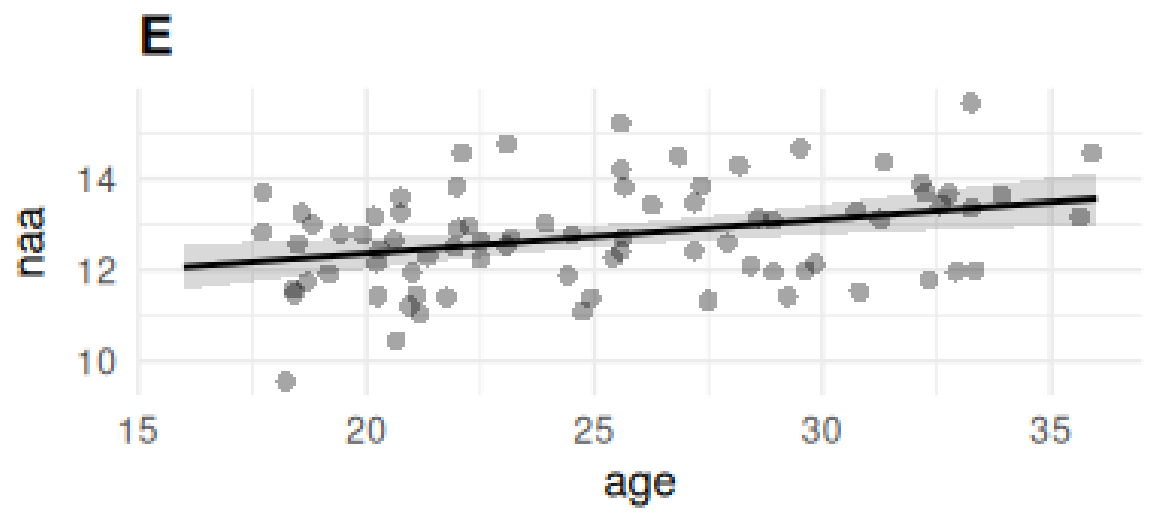

term	estimate	p.values
(Intercept)	11	1.1e-28 ****
age	0.075	0.0024 **

Figure S 2: Associations between the MRS metabolites of the mPFC with age. Both tCho and NAA showed significant associations with age (False Discovery rate corrected age p-values: myo-Ins=0.55, Glu=0.66, GSH=0.21, tCho=0.000083*, NAA=0.0060*).

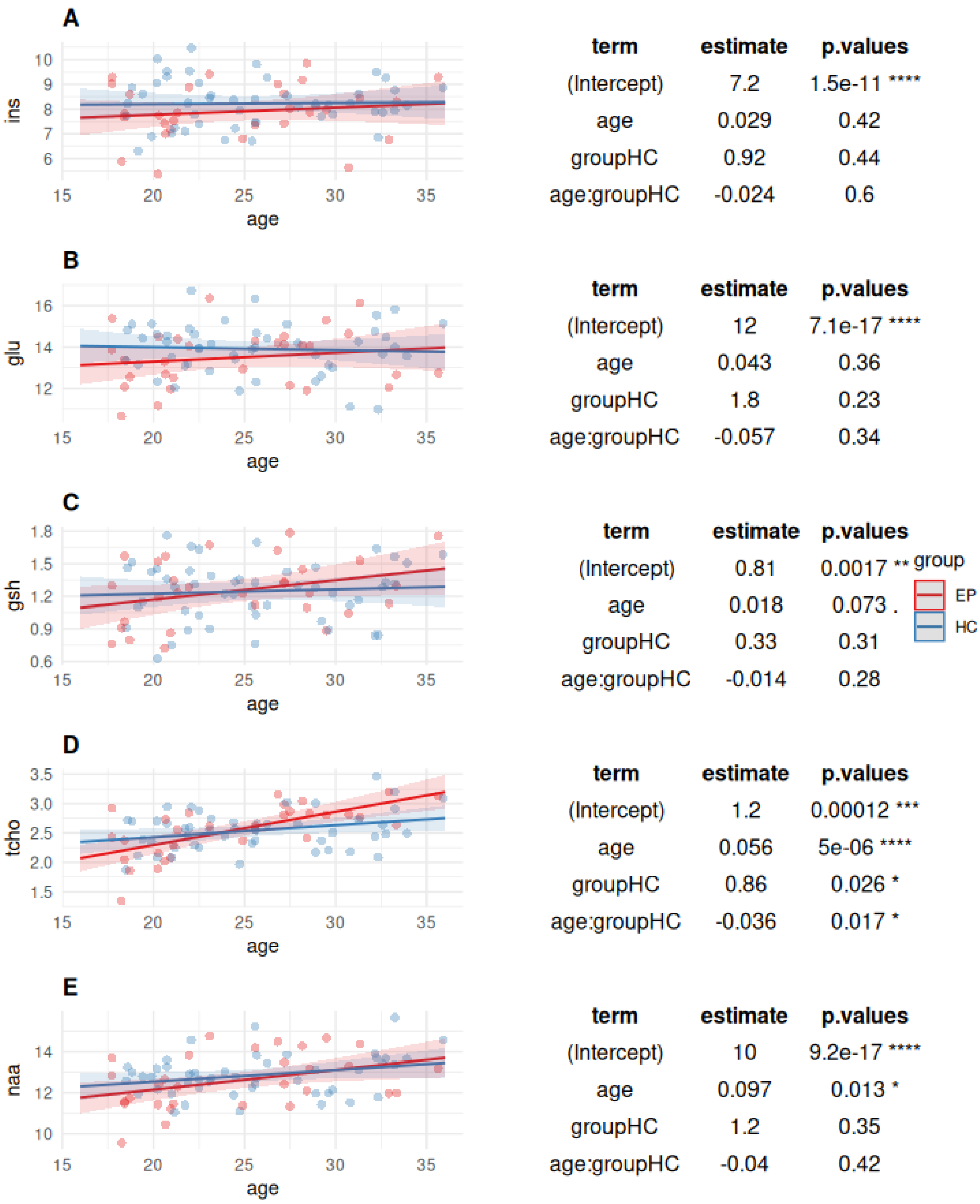

A

term	estimate	p.values
(Intercept)	7.2	1.5e-11 ****
age	0.029	0.42
groupHC	0.92	0.44
age:groupHC	-0.024	0.6

B

term	estimate	p.values
(Intercept)	12	7.1e-17 ****
age	0.043	0.36
groupHC	1.8	0.23
age:groupHC	-0.057	0.34

C

term	estimate	p.values
(Intercept)	0.81	0.0017 **
age	0.018	0.073 .
groupHC	0.33	0.31
age:groupHC	-0.014	0.28

D

term	estimate	p.values
(Intercept)	1.2	0.00012 ***
age	0.056	5e-06 ****
groupHC	0.86	0.026 *
age:groupHC	-0.036	0.017 *

E

term	estimate	p.values
(Intercept)	10	9.2e-17 ****
age	0.097	0.013 *
groupHC	1.2	0.35
age:groupHC	-0.04	0.42

Figure S 3: Interaction models between the MRS metabolites of the mPFC with age by group. Before False Discovery rate (FDR) correction, only tCho showed a significant interaction between healthy controls (HC) and age (False Discovery rate corrected age:groupHC interaction p-values: myo-Ins=0.69, Glu=0.52, GSH=0.52, tCho=0.084, NAA=0.52).

Associations between myo-ins MRS metabolites and WM microstructure

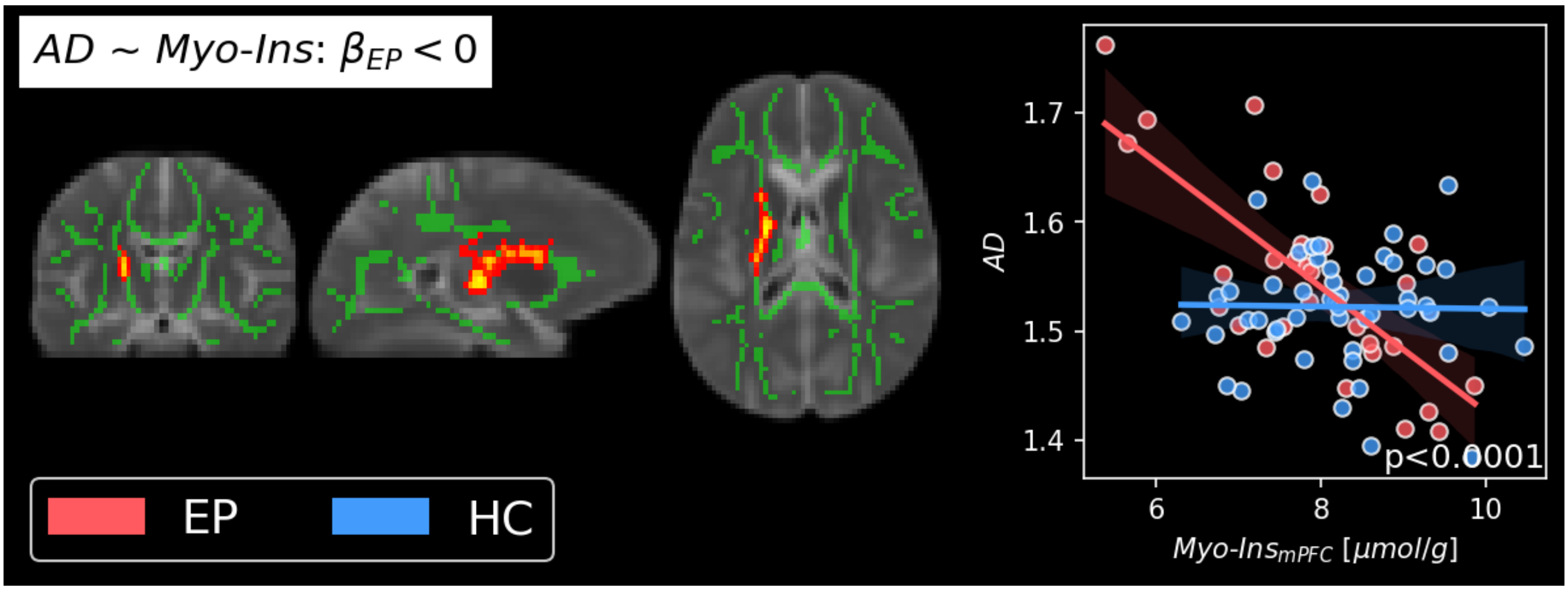

Figure S 4: Association clusters between myo-Ins of the mPFC and the microstructure metric AD. Localized in the left internal capsule, AD is lower at higher concentrations of myo-inositol in the mPFC ($myo - Ins_{mPFC}$) in EP participants. $\beta_{EP} < 0$: contrast testing the slope of EP is significantly negative. L: left hemisphere.

Post Hoc Analyses

Myo-inositol frequency mask

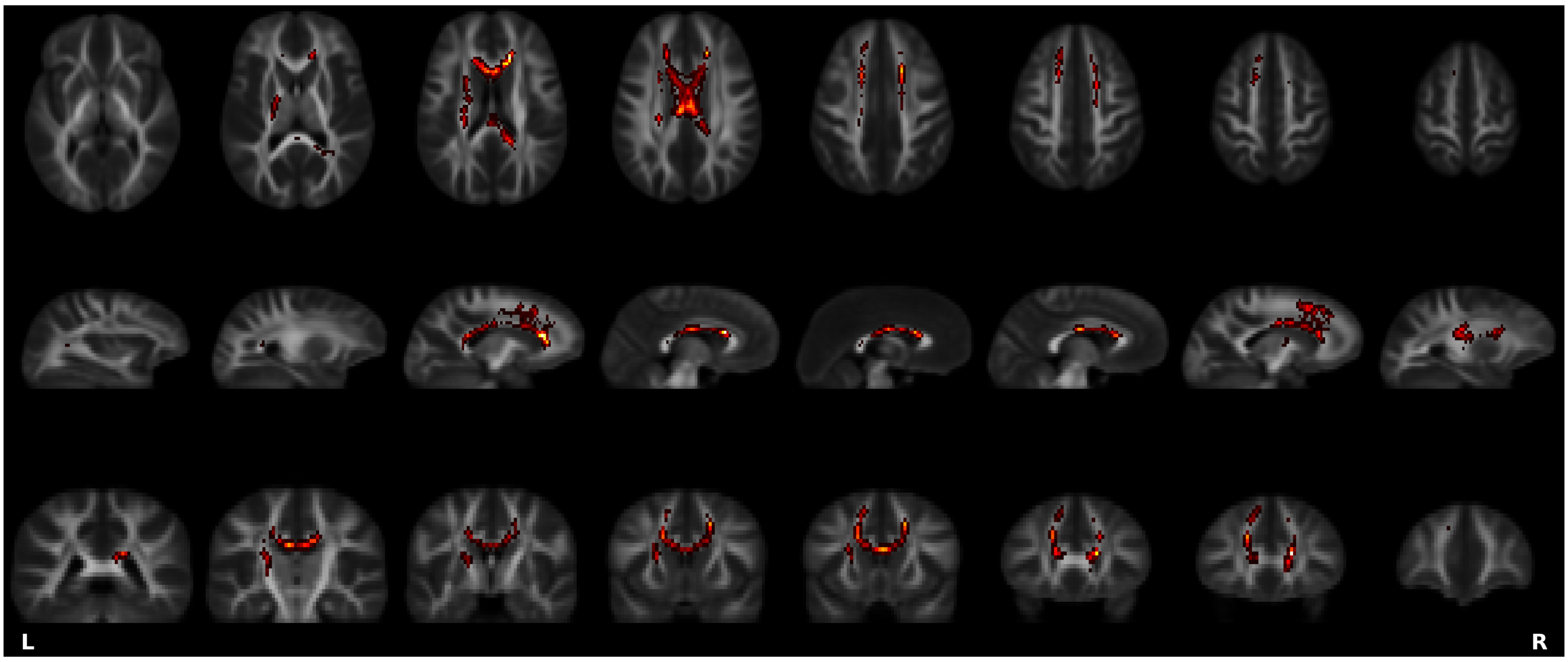

Figure S 5: WM mask used to define the cluster utilized in all post hoc analyses. The mask was generated by combining all the skeleton masks of significant WM clusters across dMRI modalities and contrasts of the myo-Ins associations. The mask comprises primarily forceps minor, region I, II and III of the corpus callosum (Hofer and Frahm (2006), i.e. genu and anterior part of the body), and anterior corona radiata. All these white matter regions contain projections and afferent fibers to the medial prefrontal cortex. Note, the mask was enlarged to aid the visualization with *tbss_fill*.

Medication, duration of illness and Substance abuse

In the literature a reduction in Glu and myo-In has been reported as a consequence of drug and/or cannabis exposure in non-psychiatric participants (Watts et al., 2020) and EP (Rigucci et al., 2018). In this cohort, no metabolites (all $p \geq 0.14$) nor WM metric (all $p \geq 0.27$) showed significant differences between EP participants exposed or not to cannabis. Similarly, alcohol and drug consumption (CMRS total ≥ 1) does not affect WM metrics ($p \geq 0.72$) or metabolites ($p \geq 0.14$) in EP, except for GSH in which those who consume have higher GSH than those who do not ($p \leq 0.03$). None of the metabolite concentrations were associated with chlorpromazine equivalent dose ($p \geq 0.12$) or duration of illness ($p \geq 0.35$). Myo-Inositol WM clusters for f and $D_{e,||}$ were not associated to chlorpromazine equivalent dose dose, $p \geq 0.16$) or duration of illness ($p \geq 0.17$).

Partial R^2

	MyoIns	Glu	GSH	tCho	NAA	Full model Adjusted R-squared
f	0.19	-0.04	0.03	0.05	-0.03	0.23
$D_{e,\|\|}$	0.28	0.02	0.02	0.09	0.03	0.36

Table S 3: Coefficients of partial determination (partial R^2), for all metabolites of both f and $D_{e,||}$ models (EQ. 2 in the main text). Partial R^2 measures the marginal contribution of a specific metabolite (e.g. myo-inositol) to the WM microstructures, indicating the proportion of variance in f or $D_{e,||}$ not explained by the other metabolites that is explained by that specific metabolite when added to the model.

Robustness analyses

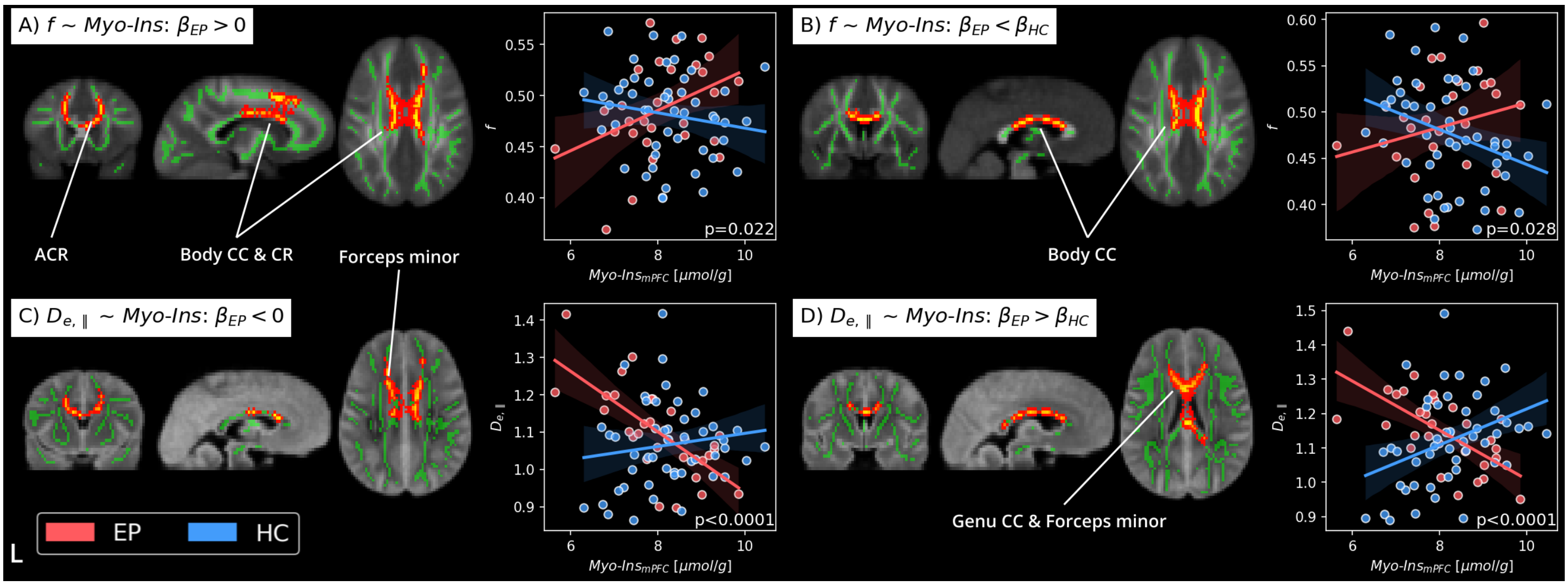

Figure S 6: TBSS clusters of associations between f and $D_{e,||}$ and myo-inositol concentration in the mPFC when EP participants with the most extreme values of f ($n_{removed} = 2$) and $D_{e,||}$ ($n_{removed} = 1$) are removed. Removing the datapoints with more extreme values ($f < 0.35$ and $D_{e,||} > 1.6$) does not qualitatively change the overall results, except for a decrease in the significance level of f. $\beta_{EP} < 0$: Contrast testing the slope of EP reveals a significantly negative result. $\beta_{EP} < \beta_{HC}$: contrast testing the difference in slopes between EP and HC is significant, L: left hemisphere.

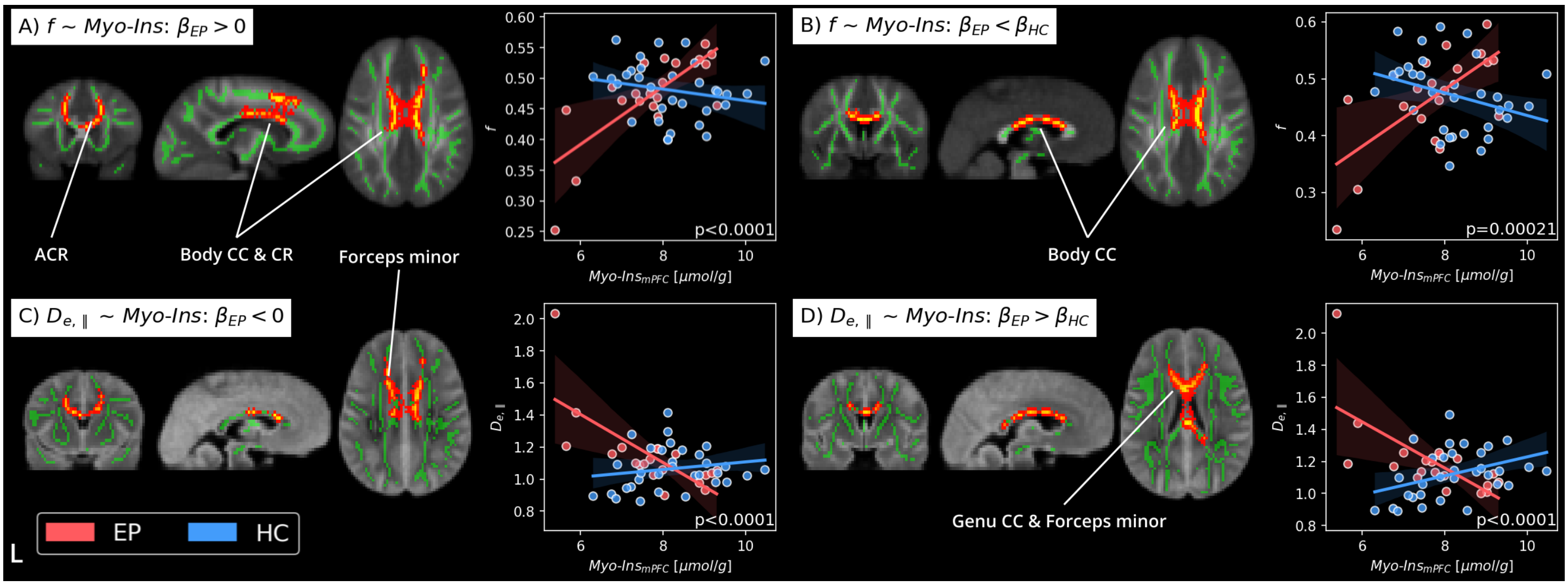

Figure S 7: TBSS clusters of associations between f and $D_{e,||}$ and myo-inositol concentration in the mPFC when female participants are removed ($n_{male} = 52$). Removing the female participants does not suppress the significant associations. $\beta_{EP} < 0$: Contrast testing the slope of EP reveals a significantly negative result. $\beta_{EP} < \beta_{HC}$: contrast testing the difference in slopes between EP and HC is significant, L: left hemisphere.

Robustness analyses

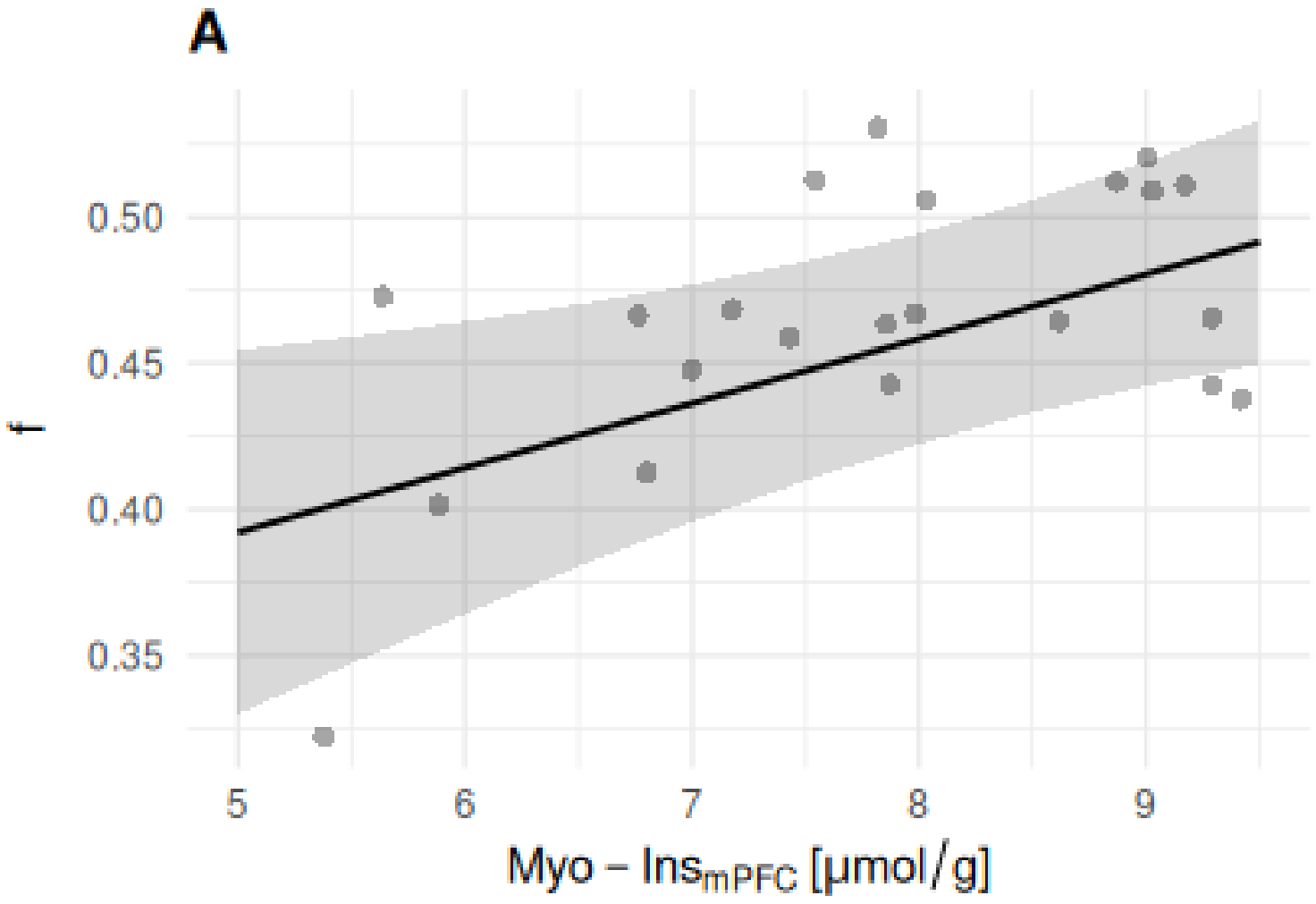

term	estimate	p.values	FDR
(Intercept)	0.26	0.0029 **	0.012 *
Myo-Ins	0.022	0.0096 **	0.019 *
Age	0.00084	0.64	0.64
Sex : Female	0.015	0.47	0.63

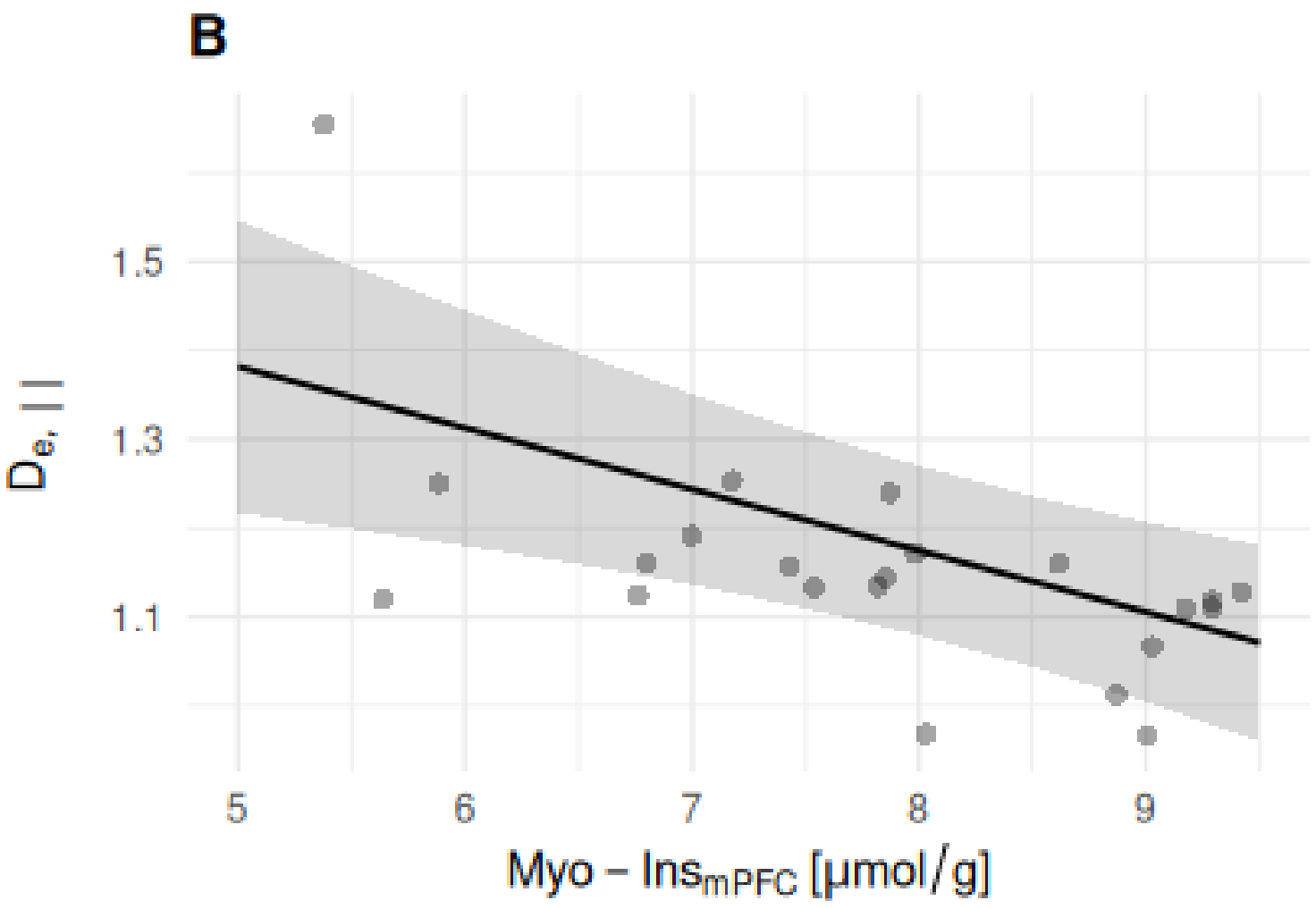

term	estimate	p.values	FDR
(Intercept)	1.8	3e-08 ****	1.2e-07 ****
Myo-Ins	-0.069	0.003 **	0.006 **
Age	-0.0049	0.31	0.4
Sex : Female	-0.047	0.4	0.4

Figure S 8: Association of EP's WM microstructure values in the post hoc cluster when patients suffering from affective psychosis are excluded (N=5 schizoaffective disorder, N=1 bipolar disorder, N=1 bipolar disorder with psychotic features, N=1 depressive disorder with psychotic features). (A) Excluding subjects suffering from affective psychosis does not suppress the association of myo-Inositol with f. (B) the myo-Ins association with $D_{e,||}$ remains qualitatively unchanged after excluding the EP participants. The regression models include a correction for age and sex. Adjusted-R2: f=0.21 and $D_{e,||}$=0.33.

References

Y. Alemán-Gómez, T. Baumgartner, P. Klauser, M. Cleusix, R. Jenni, P. Hagmann, P. Conus, K. Q. Do, M. Bach Cuadra, P. S. Baumann, and P. Steullet. Multimodal Magnetic Resonance Imaging Depicts Widespread and Subregion Specific Anomalies in the Thalamus of Early-Psychosis and Chronic Schizophrenia Patients. *Schizophrenia Bulletin*, 49(1):196–207, Jan. 2023. ISSN 0586-7614, 1745-1701. doi: 10.1093/schbul/sbac113. URL `https://academic.oup.com/schizophreniabulletin/article/49/1/196/6692485`.

J. L. R. Andersson and S. N. Sotiropoulos. An integrated approach to correction for off-resonance effects and subject movement in diffusion MR imaging. *NeuroImage*, 125:1063–1078, Jan. 2016. ISSN 1095-9572. doi: 10.1016/j.neuroimage.2015.10.019.

S. Hofer and J. Frahm. Topography of the human corpus callosum revisited—Comprehensive fiber tractography using diffusion tensor magnetic resonance imaging. *NeuroImage*, 32(3): 989–994, Sept. 2006. ISSN 1053-8119. doi: 10.1016/j.neuroimage.2006.05.044. URL `https://www.sciencedirect.com/science/article/pii/S1053811906006501`.

E. Kellner, B. Dhital, V. G. Kiselev, and M. Reisert. Gibbs-ringing artifact removal based on local subvoxel-shifts. *Magnetic Resonance in Medicine*, 76(5):1574–1581, 2016. ISSN 1522-2594. doi: 10.1002/mrm.26054. URL `https://onlinelibrary.wiley.com/doi/abs/10.1002/mrm.26054`. _eprint: https://onlinelibrary.wiley.com/doi/pdf/10.1002/mrm.26054.

H.-H. Lee, D. S. Novikov, and E. Fieremans. Removal of partial Fourier-induced Gibbs (RPG) ringing artifacts in MRI. *Magnetic Resonance in Medicine*, 86(5):2733–2750, 2021. ISSN 1522-2594. doi: 10.1002/mrm.28830. URL `https://onlinelibrary.wiley.com/doi/abs/10.1002/mrm.28830`. _eprint: https://onlinelibrary.wiley.com/doi/pdf/10.1002/mrm.28830.

S. Rigucci, L. Xin, P. Klauser, P. S. Baumann, L. Alameda, M. Cleusix, R. Jenni, C. Ferrari, M. Pompili, R. Gruetter, K. Q. Do, and P. Conus. Cannabis use in early psychosis is associated with reduced glutamate levels in the prefrontal cortex. *Psychopharmacology*, 235(1):13–22, Jan. 2018. ISSN 1432-2072. doi: 10.1007/s00213-017-4745-z. URL `https://doi.org/10.1007/s00213-017-4745-z`.

C. M. W. Tax, M. Bastiani, J. Veraart, E. Garyfallidis, and M. Okan Irfanoglu. What's new and what's next in diffusion MRI preprocessing. *NeuroImage*, 249:118830, Apr. 2022. ISSN 1053-8119. doi: 10.1016/j.neuroimage.2021.118830. URL `https://www.sciencedirect.com/science/article/pii/S1053811921011010`.

J. Veraart, D. S. Novikov, D. Christiaens, B. Ades-Aron, J. Sijbers, and E. Fieremans. Denoising of dif-

fusion MRI using random matrix theory. *NeuroImage*, 142:394–406, Nov. 2016. ISSN 1095-9572. doi: 10.1016/j.neuroimage.2016.08.016.

J. J. Watts, R. Garani, T. Da Silva, N. Lalang, S. Chavez, and R. Mizrahi. Evidence That Cannabis Exposure, Abuse, and Dependence Are Related to Glutamate Metabolism and Glial Function in the Anterior Cingulate Cortex: A 1H-Magnetic Resonance Spectroscopy Study. *Frontiers in Psychiatry*, 11:764, Aug. 2020. ISSN 1664-0640. doi: 10.3389/fpsyt.2020.00764. URL `https://www.ncbi.nlm.nih.gov/pmc/articles/PMC7468488/`.
